# Characterizing the phosphorus forms extracted from soil by the Mehlich III soil test

**DOI:** 10.1186/s12932-018-0052-9

**Published:** 2018-02-21

**Authors:** Barbara J. Cade-Menun, Kyle R. Elkin, Corey W. Liu, Ray B. Bryant, Peter J. A. Kleinman, Philip A. Moore

**Affiliations:** 1 Swift Current Research and Development Centre, Agriculture and Agri-Food Canada, Box 1030, Gate 4, Airport Drive, Swift Current, SK S9H 3X2 Canada; 2 Pasture Systems and Watershed Management Research Unit, USDA-ARS, University Park, PA 16802 USA; 3 Stanford Magnetic Resonance Laboratory, Stanford University School of Medicine & ChEM-H–Stanford University, Stanford, CA USA; 4 Poultry Production and Product Safety Research Unit, Plant Science 115, USDA-ARS, University of Arkansas, Fayetteville, AR 72701 USA

**Keywords:** Phosphorus, Alum, Phytate, Poultry litter, P-NMR, Mass spectrometry

## Abstract

Phosphorus (P) can limit crop production in many soils, and soil testing is used to guide fertilizer recommendations. The Mehlich III (M3) soil test is widely used in North America, followed by colorimetric analysis for P, or by inductively coupled plasma-based spectrometry (ICP) for P and cations. However, differences have been observed in M3 P concentrations measured by these methods. Using ^31^P nuclear magnetic resonance (P-NMR) and mass spectrometry (MS), we characterized P forms in M3 extracts. In addition to the orthophosphate that would be detected during colorimetric analysis, several organic P forms were present in M3 extracts that would be unreactive colorimetrically but measured by ICP (molybdate unreactive P, MUP). Extraction of these P forms by M3 was confirmed by P-NMR and MS in NaOH-ethylenediaminetetraacetic acid extracts of whole soils and residues after M3 extraction. The most abundant P form in M3 extracts was *myo*-inositol hexaphosphate (*myo*-IHP, phytate), a compound that may not contribute to plant-available P if tightly sorbed in soil. Concentrations of *myo*-IHP and other organic P forms varied among soils, and even among treatment plots on the same soil. Extraction of *myo*-IHP in M3 appeared to be linked to cations, with substantially more *myo*-IHP extracted from soils fertilized with alum-treated poultry litter than untreated litter. These results suggest that ICP analysis may substantially over-estimate plant-available P in samples with high MUP concentrations, but there is no way at present to determine MUP concentrations without analysis by both colorimetry and ICP. This study also tested procedures that will improve future soil P-NMR studies, such as treatment of acid extracts, and demonstrated that techniques such as P-NMR and MS are complimentary, each yielding additional information that analysis by a single technique may not provide.
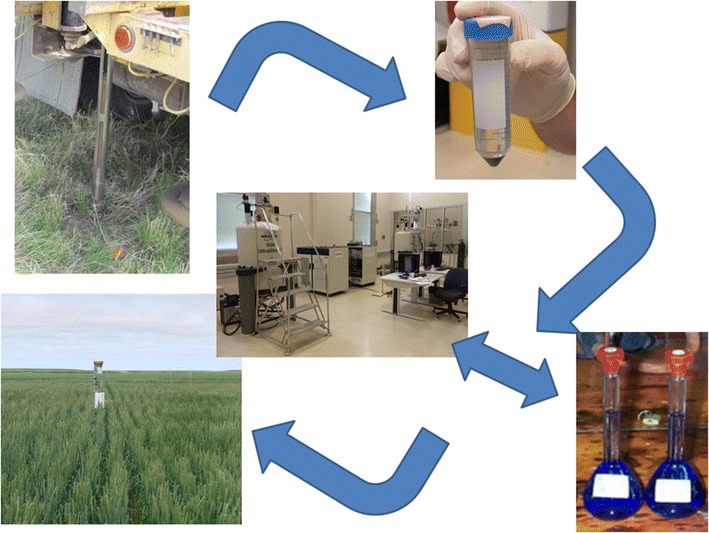

## Background

Phosphorus is an essential element that is also considered a macronutrient for agricultural crops, as it is required in relatively large quantities. Modern fertilizer recommendations take into account estimates of P in soil that is available to the crop, generally determined by chemical extracts (i.e. soil tests) that have been developed over the past six decades. Despite this long history, considerable uncertainty surrounds the interpretation of soil P tests, because P speciation in soil tests is inferred. Indeed, differences in recommendations for P fertilization from soil P tests are of particular concern in areas where soil, fertilizer and manure P contribute to water pollution [1–3].

Soil tests estimate plant-available P indirectly, supported by correlations between soil P test values and crop yields. Soil P tests seek to mimic plant uptake, extracting all or a proportional amount of the P available for plant use during the growing season, including both labile organic and inorganic P compounds. Ideally, a soil test indicates when soil P concentrations are low enough to reduce crop growth, giving an economic return on an investment in fertilizer, but will also indicate when P concentrations are in excess, such that additional P fertilization should be avoided to reduce the risk of P loss. To be of broad applicability, a soil test must also be quick, easy to implement, inexpensive, and appropriate for a wide range of soil types and conditions [4, 5].

Many soil P tests are in use world-wide, with over 13 soil P tests developed for agronomic recommendations in North America alone. Soil P test usage varies, with the selection of test typically decided by local soil conditions (e.g. some tests are better suited to high or low pH conditions than others), although historical and institutional factors also influence the choice of test in different areas [4, 6]. Historically, soil tests were all analyzed colorimetrically, such as by the molybdate blue method [7], which has been interpreted to measure orthophosphate in solution (termed molybdate-reactive P, or MRP). Although other labile organic and inorganic P compounds may be extracted from soil, colorimetric P measurements will not detect these other P forms, or may over-estimate orthophosphate if these P forms are hydrolyzed during the extraction and determination processes [8].

Following the advent of inductively coupled plasma-based spectrometry (ICP), new soil tests were developed in the 1970s and 1980s to enable the simultaneous measurement of multiple elements from a single soil extract [9–12]. Unlike colorimetry, ICP determines total P in solution, which includes both MRP and molybdate-unreactive P (MUP), with MUP being the difference between ICP-determined P and MRP. Higher P concentrations in M3 extracts with ICP analysis than with colorimetric analysis have been widely reported, suggesting MUP is common in M3 extracts [9–16].

Today, the most widely adopted multi-elemental soil P test designed for ICP is the Mehlich III (M3) test [17], the use of which is expanding across the USA and Canada [9, 11]. The M3 extractant combines acetic acid (CH_3_COOH), ammonium nitrate (NH_4_NO_3_), ammonium fluoride (NH_4_F), nitric acid (HNO_3_) and ethylenediaminetetraacetic acid (EDTA) at pH 2.5 to determine both soil test P and exchangeable cations, and can be used over a wider range of soil pH than other soil tests. In addition, the M3 test has been used as an environmental P test in many regions, due to strong correlations between M3 P and dissolved P in runoff as well as good correspondence between soil P sorption saturation estimated by M3 P, Fe and Al with soil P sorption saturation measured by other extraction methods [18, 19].

Notably, differences have been observed in M3 P measured by colorimetry and by ICP. For example, Huang et al. [15, 16] reported much larger MUP concentrations in M3 extracts of soils fertilized with poultry litter stabilized with alum [(Al_2_SO_4_)_3_·14H_2_O] than in M3 extracts from soils fertilized with untreated poultry litter. However, studies across a range of soils show no clear patterns for MUP concentrations in M3 extracts; concentrations were not consistently higher with ICP analysis than with colorimetric analysis, and they were not consistently associated with soil properties such as pH, organic matter or cations [11, 13, 14]. Some authors have suggested that differences in P concentrations between colorimetry and ICP are due to extraction of organic P in addition to orthophosphate [10, 11, 13]. Phosphorus forms differ in their bioavailability and environmental reactivity [20]; if M3 extraction with ICP analysis is to be used for meaningful fertilizer or environmental recommendations, it is important to identify all the forms removed from soil during extraction, and to know if the extracted compounds will vary among soil types or with amendments such as manures. Although the extraction of organic P, specifically *myo*-inositol hexaphosphate (*myo*-IHP, phytate) by M3 was studied in model systems [21], we are unaware of any studies that have characterized P forms in M3 extracts from soil.

Solution ^31^P nuclear magnetic resonance spectroscopy (P-NMR) is the most widely used method to characterize P forms in extracts of soils and other environmental samples [5, 22, 23]. The current standard method to extract P from soils for NMR is NaOH-EDTA, with analysis at pH > 12 to give optimal peak separation and consistency in chemical shifts, which are pH-dependent in P-NMR [22, 24, 25]. However, adjusting sample pH for optimal P-NMR may cause problems with acid extracts from some sample types. Although pH-adjusted acid extracts have been successfully used for P-NMR as components of sequential fractionation of animal manures and have shown a range of P compounds to be present [26, 27], only orthophosphate was detected in acid extracts of soils in previous studies [27, 28]. While orthophosphate may be the only P form in those extracts, it is also possible that other P forms were lost by precipitation with aluminum (Al) and iron (Fe) when the pH of soil extracts was increased, because these metals are found in much higher concentrations in soils than in animal manures. One approach that was recently used successfully to determine the effect of oxalate extraction on soil organic P forms was to treat acidic oxalate extracts with a cation exchange resin prior to pH adjustment, and to extract soil residues after oxalate extraction with NaOH-EDTA, comparing the changes in soil P forms to those from whole soil samples extracted in NaOH-EDTA and in the oxalate extracts [29]. Combining several spectroscopic techniques to analyze extracts can also confirm compound identifications [23].

Characterizing all P compounds in M3 extracts is essential to understanding potential differences in agronomic and environmental recommendations derived from variations in analytical procedures. As such, the objective of this study was to determine differences in P forms extracted from soils with different cation profiles, by characterizing P compounds in M3 extracts and in NaOH-EDTA extracts before and after M3 extraction. In particular, we sought to explain differences in colorimetric and ICP measurements of M3 P. To do so, M3 extracts were analyzed by P-NMR to identify all P compounds in extracts, and by mass spectrometry (MS) for inositol hexaphosphate concentrations.

## Methods

### Soil samples

This study originated as two independent projects investigating P forms in M3 extracts that were merged into a single study; as such, soils from two different sources were used. Four samples were selected from a 20-year research project at the Main Agricultural Experiment Station of the University of Arkansas in Fayetteville, AR. These are described in detail elsewhere [15, 30, 31]. Soils from these studies were specifically selected to have the greatest contrast in the M3 P values between ICP and colorimetry, to yield the highest MUP concentrations, based on prior research [e.g. 15]. Surface soil (0–5 cm) samples were taken from grassed [tall fescue, *Festuca arundinacea* (Schreb.) Dumort., nom. cons.] plots from four treatments: an unfertilized control, and plots receiving alum-treated poultry litter, untreated poultry litter at 8.96 Mg ha^−1^, or ammonium nitrate at 260 kg N ha^−1^. In addition, three Canadian soils that had been previously characterized by P-NMR were also used. One sample (SK), was collected from a long-term continuous wheat plot at the Agriculture and Agri-Food Canada (AAFC) Swift Current Research and Development Centre in Saskatchewan Canada [32], a second soil (PEI) was from a long-term research plot at the AAFC Charlottetown Research and Development Centre in Prince Edward Island, Canada [33], and one sample was a glacial till reference soil (Till-1) that was collected near Lanark, Ontario, Canada, which was purchased from the Canadian Certified Reference Materials Project of Natural Resources Canada. Prior analysis indicated that these soils varied in their P forms and cation profiles. Details about the soil samples are given in Table 1.

**Table 1:** General information about the soils used in this study

	AR-control	AR-N	AR-PL	AR-PL-Alum	SK	PEI	Till Ref
Source	USDA-ARS plots, Fayetteville, AR USA				AAFC, SK Canada	AAFC, PEI, Canada	CCRMP, NRC, Canada
Treatment	Control soil	Commercial N treatment	Untreated poultry litter	Alum-treated poultry litter	Continuous wheat	No till	Commercial Reference Material
Depth, cm	0–5	0–5	0–5	0–5	0–7.5	0–5	B, C horizons
pH (H_2_0)	5.7	4.8	6.3	5.4	5.9	4.7	5.9
Total N, %	0.17	0.21	0.25	0.24	0.20	0.17	0.20
Total C, %	1.68	2.03	2.39	2.06	2.09	2.29	2.03
Total P, mg kg^−1^	473	470	1345	1733	548	1097	930
Organic P, mg kg^−1^ (%)^a^	241 (51)	211 (45)	216 (16)	730 (42)	278 (51)	407 (36)	374 (40)
Organic P, mg kg^−1^ (%)^b^	144 (51)	177 (54)	165 (22)	483 (41)	177 (52)	175 (23)	139 (39)
References	[15, 30, 39]				[31]	[32]	

^a^Determined by the ignition method

^b^Determined by P-NMR spectroscopy

### Sample extraction

**Fig. 1:**
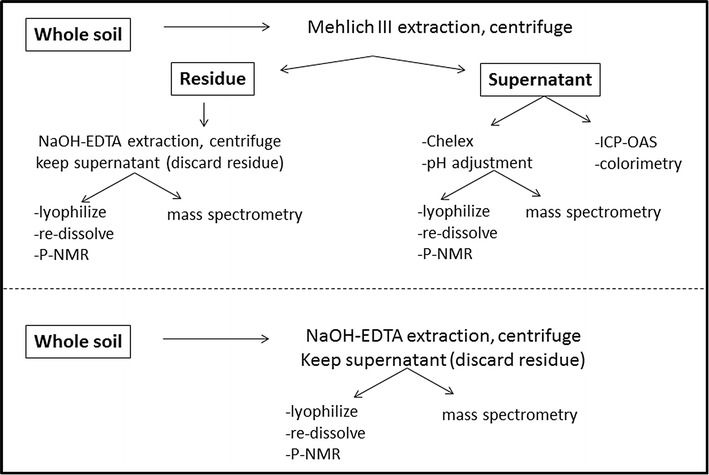
A flow chart of the extraction and analysis procedures used in this study. Please see the text for a full description of the “Methods”

Duplicate samples were extracted in the same way for analysis by P-NMR or MS (Fig. 1).

#### Mehlich III

Samples were extracted for 5 min in M3 extract with an end-over-end shaker, using 4 g soil in 40 mL. Samples were centrifuged for 8 min at 12,000×*g* at 20 °C, the supernatant was filtered with a 0.2 µm syringe filter, and 10 mL was put aside for colorimetric analysis for MRP [7] and ICP-optical emission spectrometry (OES) analysis for P, Al, Fe, calcium (Ca), magnesium (Mg) and manganese (Mn). The residues were washed with ~ 10 mL of deionized water, shaken by hand for ~ 30 s, centrifuged as described, and the supernatants (~ 8 mL) were added to the M3 extracts after syringe filtering as described. The filtered M3 extracts were shaken (end-over-end shaker) for 5 min with 1 g analytical grade Chelex 100 cation exchange resin (Bio-Rad Laboratories, Hercules, CA) to remove cations, particularly Fe and Al, and then vacuum filtered (Whatman 42) to remove the resin. The filtrate pH was subsequently adjusted from ~ 3.5 to ~ 12 with a solution of 2.5 mol L^−1^ NaOH + 0.5 mol L^−1^ Na_2_EDTA (15 mol L^−1^ NH_4_OH was used to adjust the pH in the MS extractions). Prior tests showed that adjusting the solution pH without first using Chelex caused P to precipitate from solution with cations.

#### NaOH-EDTA

Whole soil samples (3 g) and the washed M3 residues were extracted with a modified version of the Cade-Menun and Preston method [34]: 30 mL of 0.25 mol L^−1^ NaOH + 0.05 mol L^−1^ Na_2_EDTA (NaOH-EDTA) for 4 h at room temperature in the dark with an end-over-end shaker, followed by centrifugation (20 min, ~ 12,000×*g*). A 1-mL aliquot was removed, diluted to 10 ml with deionized water, and analyzed by ICP-OES for P, Fe and Mn concentrations.

The NaOH-EDTA supernatants and pH-adjusted M3 extracts to be analyzed by P-NMR were placed in 50 mL disposable centrifuge tubes, frozen (− 20 °C, 48 h) and lyophilized.

### P-NMR spectroscopy

#### Sample preparation and analysis

All the lyophilized material for each sample was dissolved in 0.65 mL each of D_2_O and H_2_O, 1.35 mL of the NaOH-EDTA extracting solution and 0.8 mL of 10 M NaOH. Dissolved samples were intermittently vortexed over a period of ~ 5 min, centrifuged (1500×*g*, 20 min), and supernatant transferred to 10 mm NMR tubes for P-NMR analysis. Samples not immediately analyzed by NMR were stored in a refrigerator and analyzed within 24 h.

All extracts of the AR samples were analyzed at the Stanford Magnetic Resonance Laboratory (SMRL, Stanford University) using a Varian INOVA 600 MHz spectrometer; all extracts of the other samples were analyzed at the Saskatchewan Structural Sciences Centre (SSSC, University of Saskatchewan) with a Bruker Avance 500-MHz spectrometer. Both spectrometers were equipped with 10-mm broadband probes. The NMR experimental parameters were: 45° pulse width (13 µs at SSSC, 23 µs at SMRL), 0.675 s acquisition, 4.32 s delay, and no proton decoupling. This delay was estimated to be sufficient based on the ratio of P/(Fe + Mn) in the NaOH-EDTA extracts [22, 35], but may have been too short for fully quantitative analysis for the Mehlich extracts if Fe precipitated out when preparing lyophilized samples. For the NaOH-EDTA extracts of whole soils and residues, 2900 scans were acquired (4 h); 5800 scans were acquired (8 h) for the M3 extracts, due to lower sample P concentrations.

#### Peak identification

**Fig. 2:**
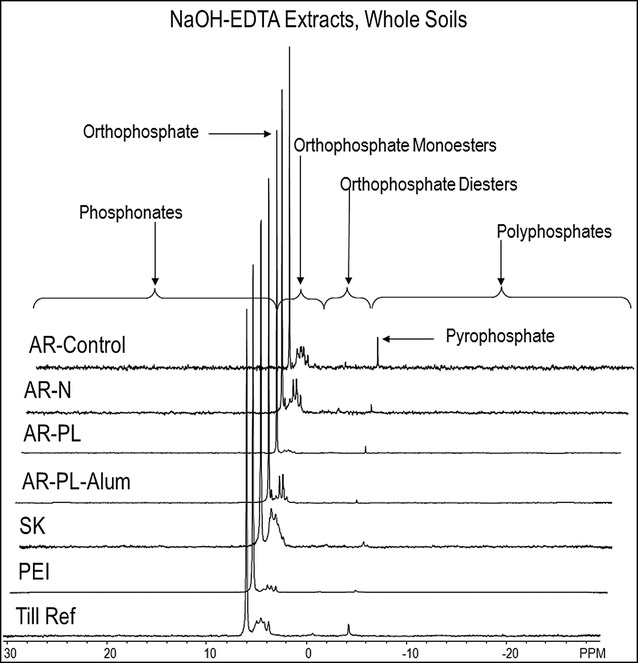
P-NMR spectra of whole soil samples extracted with NaOH-EDTA. Spectra are plotted with 7 Hz line-broadening and scaled to the height of the orthophosphate peak

**Fig. 3:**
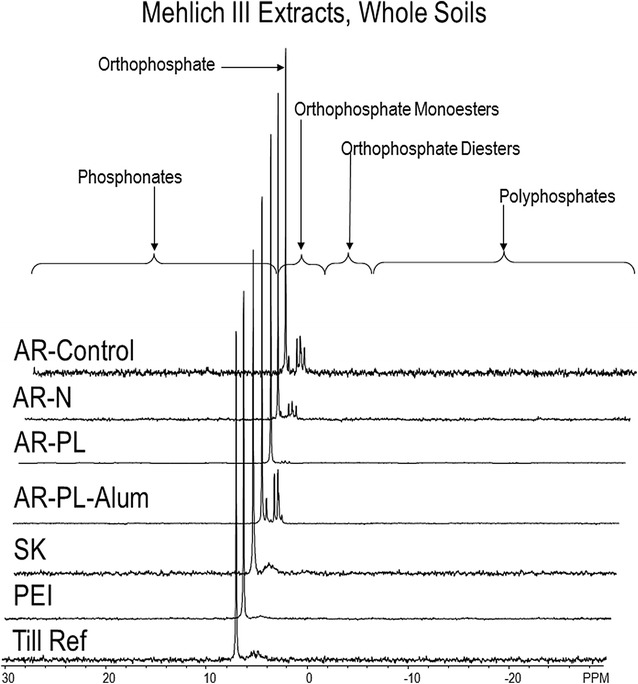
P-NMR spectra of whole soil samples extracted with Mehlich III solution. Spectra are plotted with 7 Hz line-broadening and scaled to the height of the orthophosphate peak

**Fig. 4:**
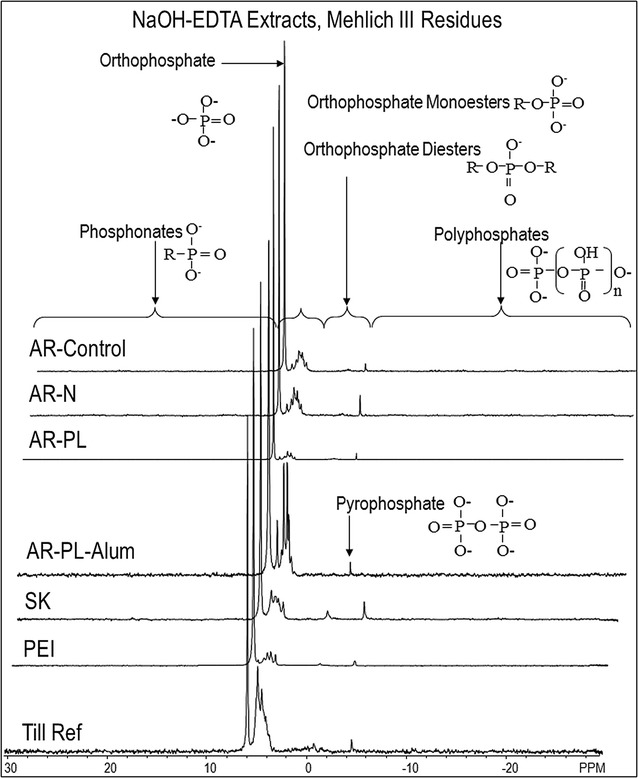
P-NMR spectra of Mehlich III residue soils extracted with NaOH-EDTA. Spectra are plotted with 7 Hz line-broadening and scaled to the height of the orthophosphate peak

**Fig. 5:**
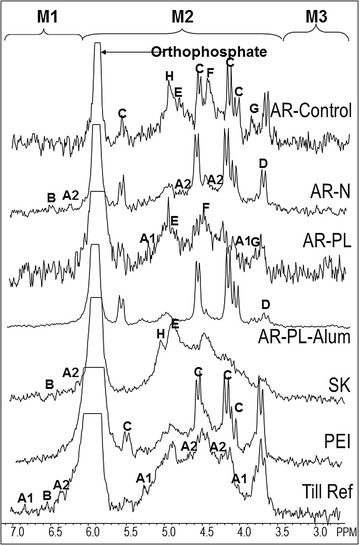
Enhanced orthophosphate monoester region of P-NMR spectra of whole soil samples extracted with NaOH-EDTA. Spectra are processed with 2 Hz line-broadening, and are scaled to the tallest peak in the M2 region. A1 and A2, *chiro*-inositol hexaphosphate (IHP) in the 4-equatorial, 2-axial 4-axial, 2-equatorial conformations, respectively; B, *neo*-IHP; C, *myo*-IHP; D, *scyllo*-IHP; E, α-glycerophosphate; F, β-glycerophosphate; G, choline phosphate; H, unidentified peak at 5 ppm. Note that not all peaks are labelled in all spectra in the figure

Spectra are shown in Figs. 2, 3, 4 and 5. Chemical shifts were determined relative to an external orthophosphoric acid standard (85%). Signals were assigned to P compounds based on the literature after standardizing the orthophosphate peak to 6 ppm [25, 33] and by spiking selected samples with reference compounds (β-glycerophosphate, P choline and *myo*-IHP; [25]). Peak areas were calculated by integration and manual measurement on spectra processed with 2 and 7 Hz line broadening, using NMR Utility Transform Software (NUTS, Acorn NMR, Livermore CA; 2006 edition). There were small variations in chemical shift among the different extracts (Table 2). Three groups of inorganic P were detected: orthophosphate at 6.00 ppm, pyrophosphate at − 4.06, and polyphosphates between − 4.0 and − 25.0 ppm. Organic P compound classes included phosphonates from 30.0 to 7.15 ppm, orthophosphates monoesters from 6.9 to 6.2 ppm and at 5.9 to 2.7 ppm, and orthophosphates diesters between 2.7 and − 3.6 ppm. Each of these organic P classes contained a number of specific P forms, with the chemical shifts shown in Table 2. One peak at ~ 5.0 ppm appears to correspond with the recently identified broad high-molecular weight P [36], based on separate tests (Cade-Menun, unpublished data), and thus was identified and quantified in spectra. Other peaks in the monoester region not specifically identified were grouped into the Mono 1, Mono 2 and Mono 3 categories. For these, the area of the total region was determined, and then the areas of the specifically identified peaks in these regions were subtracted. For the diesters, only deoxyribonucleic acid (DNA) was specifically identified; the remaining peaks were grouped into the category Other diesters based on chemical shift. Results were corrected for diester degradation products by subtracting the peak areas of α-glycerophosphate, β-glycerophosphate, and all mononucleotides from the orthophosphate monoester concentration and adding them to the orthophosphate diester concentration [37, 38]. Concentrations of P forms were determined by multiplying peak areas by the TP concentration of each extract (Table 3).

**Table 2:** Chemical shifts of peaks detected in P-NMR spectra

Category	P form or compound class	Chemical shift (ppm)
Inorganic P		
	Orthophosphate	6.00 ± 0.00
	Pyrophosphate	− 4.06 ± 0.37
	Polyphosphates	− 4.02 ± 0.32, − 4.89 ± 0.55, − 6.80 ± 0.79, − 8.95 ± 0.86, − 11.98 ± 0.83, − 16.68 ± 0.82, − 19.01 ± 0.89, − 20.99 ± 0.66, − 24.97 ± 0.74
Organic P		
	Phosphonates	29.89 ± 0.75, 27.32 ± 0.67, 20.40 ± 0.10, 18.81 ± 0.19, 14.97 ± 0.48, 12.75 ± 0.70, 10.00 ± 0.95, 8.10 ± 0.23, 7.35 ± 0.17
Orthophosphate monoesters		
	*myo*-IHP	5.49 ± 0.21, 4.56 ± 0.18, 4.16 ± 0.19, 4.06 ± 0.22
	*scyllo*-IHP	3.71 ± 0.17
	*neo*-IHP	6.43 ± 0.15, 4.26 ± 0.19
	d*-chiro*-IHP 4e/2a	6.59 ± 0.17, 5.29 ± 0.18, 3.96 ± 0.20
	d*-chiro*-IHP 4a/2e	6.21 ± 0.19, 4.84 ± 0.20, 4.31 ± 0.17
	Choline phosphate	3.85 ± 0.18
	Unknown	5.01 ± 0.15
	Monoester 1	6.86 ± 0.06, 6.69 ± 0.06, 6.37 ± 0.14, 6.24 ± 0.20
	Monoester 2	5.82 ± 0.07, 5.69 ± 0.08, 5.50 ± 0.17, 5.29 ± 0.12, 4.83 ± 0.14, 4.67 ± 0.15
	Monoester 3	3.71 ± 0.06, 3.53 ± 0.07, 3.32 ± 0.10, 3.12 ± 0.10, 2.96 ± 0.10, 2.72 ± 0.11
Degradation compounds		
	α-Glycerophosphate	4.85 ± 0.02
	ß-Glycerophosphate	4.67 ± 0.02
	Mononucleotides	4.53 ± 0.06, 4.46 ± 0.11, 4.37 ± 0.14, 4.267 ± 0.14, 4.10 ± 0.12
Orthophosphate diesters		
	DNA	− 0.78 ± 0.26, − 0.91 ± 0.32
	Other diesters	2.35 ± 0.11, 1.96 ± 0.10, 1.55 ± 0.17, 1.15 ± 0.22, 0.65 ± 0.19, 0.33 ± 0.27, 0.07 ± 0.19, − 0.27 ± 0.20, − 0.57 ± 0.23, − 1.14 ± 0.21, − 1.52 ± 0.09, − 1.79 ± 0.13, − 2.15 ± 0.22, − 2.55 ± 0.23, − 3.01 ± 0.18, − 3.63 ± 0.21

Peaks present in at least 10 of the 21 samples analyzed for the project, with all extractions grouped together

*IHP* inositol hexakisphosphate, *4e/2a* phosphates arranged in the 4-equatorial, 2-axial conformation, *4a/2e* phosphates arranged in the 4-axial, 2-equatorial conformation

**Table 3:** Concentrations of P and cations in each extract for each soil

	Extractant		AR-control	AR-N	AR-PL	AR-PL-alum	SK	PEI	Till Ref
TP	Mehlich III	Soil	64 (13)	84 (16)	382 (32)	675 (55)	99 (18)	244 (22)	30 (3)
	NaOH-EDTA	Residue	255 (53)	306 (58)	601 (50)	1101 (90)	204 (36)	396 (36)	328 (35)
	NaOH-EDTA	Soil	282 (59)	327 (62)	753 (63)	1178 (97)	340 (60)	759 (69)	360 (39)
	NaOH-EDTA	Difference	27 (6)	21 (4)	152 (13)	77 (7)	136 (24)	363 (33)	32 (4)
MUP	Mehlich III	Soil	33 (53)	25 (33)	4 (< 1)	306 (41)	33 (39)	33 (14)	1 (3)
Al	Mehlich III	Soil	421	695	506	1205	671	1256	1683
Fe	Mehlich III	Soil	106	220	160	161	146	216	335
Mn	Mehlich III	Soil	112	54	113	32	174	29	179
Ca	Mehlich III	Soil	1152	376	1547	853	1548	441	972
Mg	Mehlich III	Soil	148	49	266	112	347	53	161

Concentrations are given in mg kg^−1^ soil. MUP, concentration of molybdate-unreactive P, determined as the difference in P measured by ICP and colorimetrically (percent of total soil P recovered in M3 extract). All other concentrations determined by ICP-OES. Residue, residual soil after Mehlich III extraction; TP, total P in each extract (percent of total soil P recovered in each extract). Difference is the difference in between the NaOH-EDTA extracts of whole soils and the NaOH-EDTA extracts of the residue after Mehlich III extraction

### Mass spectrometry

Sample extracts were directly injected into a Q Exactive Orbitrap MS (Thermo-Fisher Scientific, Bremen, Germany) using a heated electrospray injection (HESI) source operated in negative ion mode. The sample was introduced into the HESI chamber using a Fusion 101 syringe pump (Chemyx Inc., Stafford, TX), at a rate of 25 µL min^−1^.

Optimal conditions were set as follows for the MS: scan range, 75–700 m/z; resolution, 70,000 full width at half maximum (FWHM); max inject time (IT) 250 ms; automatic gain control (AGC) target, 1 × 10^6^; sheath gas, 21 psi; auxiliary gas, 5 psi; sweep gas, 1 psi; spray voltage, 0.5 kV; capillary temperature, 250 °C; S-lens radio frequency, 50; auxiliary gas heater, 175 °C [39, 40]. In source collision-induced dissociation (CID), 15 eV was used to fragment the inositol phosphates in subsequent scans [41].

**Fig. 6:**
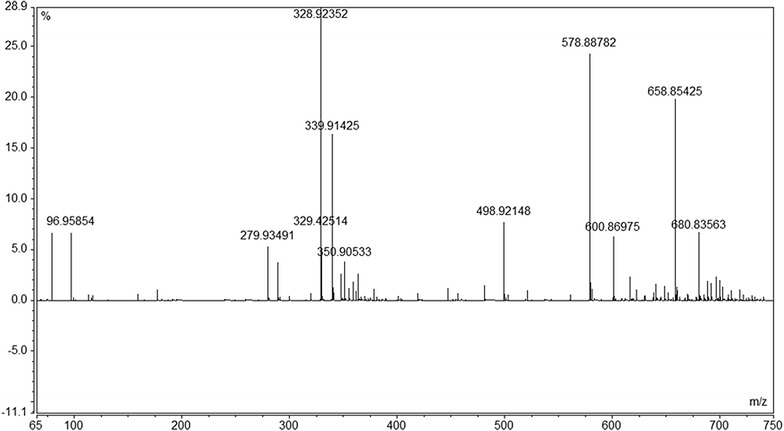
Mass spectrometry spectrum of typical sample extracted with NaOH-EDTA followed by Chelex cation removal. Most of the peaks listed in Table 7 are evident here. The 328.92 peak is the most abundant (100%), followed by 578.89 and 658.85 all of which are inositol hexaphosphates. The peaks at 96.96 and 78.85 are phosphates, which have been liberated under the 15 eV CID added in the electrospray chamber

To minimize the introduction of MS-incompatible salts, the samples were diluted 1000:1 with 18.2 MΩ water produced in-house. In some cases, an exclusion mass of 291.0828 was added prior to analysis to remove excess matrix EDTA. During integration, a three-point Gaussian smoothing algorithm was applied to quantify the data. The LC–MS system was controlled by Chromeleon 7.2 software (Thermo-Fisher Scientific, Sunnyvale CA), which was also used for data collection and processing. Once infused, signal intensities were collected over a 30 s window (approximately 50–80 scans) after the spray and total ion current were stable. Once the phytate peak was identified, the 328.92341 peak was scanned using selected ion monitoring (SIM) over a 4 Da window from 327 to 331 m/z with a resolution of 140,000 FWHM. This peak intensity was averaged over 50–80 scans (approximately 60 s) and compared to standards made up in extract matrix. The intensities of the unknowns were compared to a standard curve to determine the relative concentration of IHP in each sample. A spectrum of a typical sample in NaOH-EDTA is shown in Fig. 6, and fragmentation ions are shown in Table 4.

**Table 4:** Mass spectrometry fragmentation ions

Ion	Loss	m/z
C_6_H_17_O_24_P_6_^1−^	Parent ion (H^−^)	658.8536
C_6_H_16_O_24_P_6_^2−^	Parent ion (H^−^)	328.9234
C_6_H_16_O_21_P_5_^1−^	–HPO_3_^−^	578.8874
C_6_H_15_O_18_P_4_^1−^	(2) PO_3_^−^	498.9214
C_6_H_16_O_21_P_5_^2−^	HPO_3_^−^	288.9402
H_2_PO_4_^−^	Ejected ion	96.9585
PO_3_^−^	Ejected ion	78.9585
C_6_H_15_MgO_24_P_6_^1−^	–2H^−^	680.8356
C_6_H_14_MgO_21_P_5_^1−^	–2H^−^ + HPO_3_^−^	600.8698
C_6_H_16_NaO_24_P_6_^2−^	–2H^−^	339.9142
C_6_H_14_NaKO_18_P_4_^2−^	–2HPO_3_^−^	279.9337

## Results and discussion

### Mehlich III extraction

The soil samples used for this study had a range of pH values, and concentrations of carbon (C), nitrogen (N) and P that would be representative of many North American agricultural soils. The total M3 P concentrations ranged from 30 to 675 mg kg^−1^, representing 3–55% of soil total P concentrations (Tables 1, 3). These values are comparable to those reported in previous studies using the AR, SK and PEI soils [15, 32, 33], and are comparable to reported ranges of M3 P concentrations determined by ICP in other studies [14, 42]. The colorimetrically-determined MRP concentrations in the M3 extracts ranged from 29 to 378 mg kg^−1^, representing 47–100% of the P concentrations determined by ICP (Table 5). This is also consistent with previous studies [9–12, 14–16]. However, previous reports have suggested that the differences between colorimetry and ICP are greater at lower concentrations [10, 12, 13]. This was not consistent with the results for this study, where there was no clear trend for high or low P concentrations. The concentrations of MUP in the samples ranged from 4 to 306 mg kg^−1^, representing < 1–53% of P in the M3 extracts (Table 3).

**Table 5:** Concentrations and percentages of inorganic P compounds in extracts of whole soil or residues, determined by solution P-NMR spectroscopy

	Extractant		AR-control	AR-N	AR-PL	AR-PL-alum	SK	PEI	Till Ref
MRP	Mehlich III	Soil	31 (47)	59 (67)	378 (100)	369 (59)	60 (61)	211 (86)	29 (97)
Orthophosphate	Mehlich III	Soil	31 (49)	50 (59)	332 (87)	338 (50)	69 (70)	213 (87)	22 (72)
	NaOH-EDTA	Residue	149 (59)	157 (51)	426 (71)	497 (45)	102 (50)	275 (69)	102 (31)
	NaOH-EDTA	Soil	118 (42)	139 (43)	562 (75)	663 (56)	155 (46)	573 (76)	207 (58)
	NaOH-EDTA	Difference	− 31 (− 17)	− 17 (− 9)	136 (4)	167 (11)	54 (− 4)	298 (6)	105 (26)
Pyrophosphate	Mehlich III	Soil	0 (0)	0 (0)	1 (0)	1 (0)	0 (0)	0 (0)	0 (0)
	NaOH-EDTA	Residue	3 (1)	10 (3)	6 (1)	8 (1)	6 (3)	6 (2)	6 (2)
	NaOH-EDTA	Soil	12 (4)	7 (2)	12 (2)	20 (2)	3 (1)	5 (1)	8 (2)
Polyphosphate	Mehlich III	Soil	1 (2)	1 (1)	2 (1)	4 (1)	2 (2)	2 (1)	1 (2)
	NaOH-EDTA	Residue	2 (1)	5 (2)	2 (0)	8 (1)	4 (2)	2 (1)	4 (1)
	NaOH-EDTA	Soil	8 (3)	5 (1)	14 (2)	12 (1)	5 (1)	6 (1)	5 (1)
Total inorganic P	Mehlich III	Soil	32 (49)	50 (60)	334 (87)	343 (51)	71 (72)	215 (88)	22 (74)
	NaOH-EDTA	Residue	155 (61)	172 (56)	435 (72)	512 (47)	112 (55)	284 (72)	112 (34)
	NaOH-EDTA	Soil	138 (49)	150 (46)	588 (78)	695 (59)	163 (48)	584 (77)	221 (61)
	NaOH-EDTA	Difference	− 16 (− 12)	− 21 (− 10)	154 (6)	183 (13)	51 (7)	300 (5)	109 (27)

Concentrations are given in mg kg^−1^ soil (% of total P in each extract). MRP is molybdate-reactive P, determined colorimetrically in the Mehlich III extracts. Residue is the residual soil after Mehlich III extraction. Total inorganic P is the sum of orthophosphate, pyrophosphate and polyphosphate. Difference is the difference in between the NaOH-EDTA extracts of whole soils and the NaOH-EDTA extracts of the residue after Mehlich III extraction

**Fig. 7:**
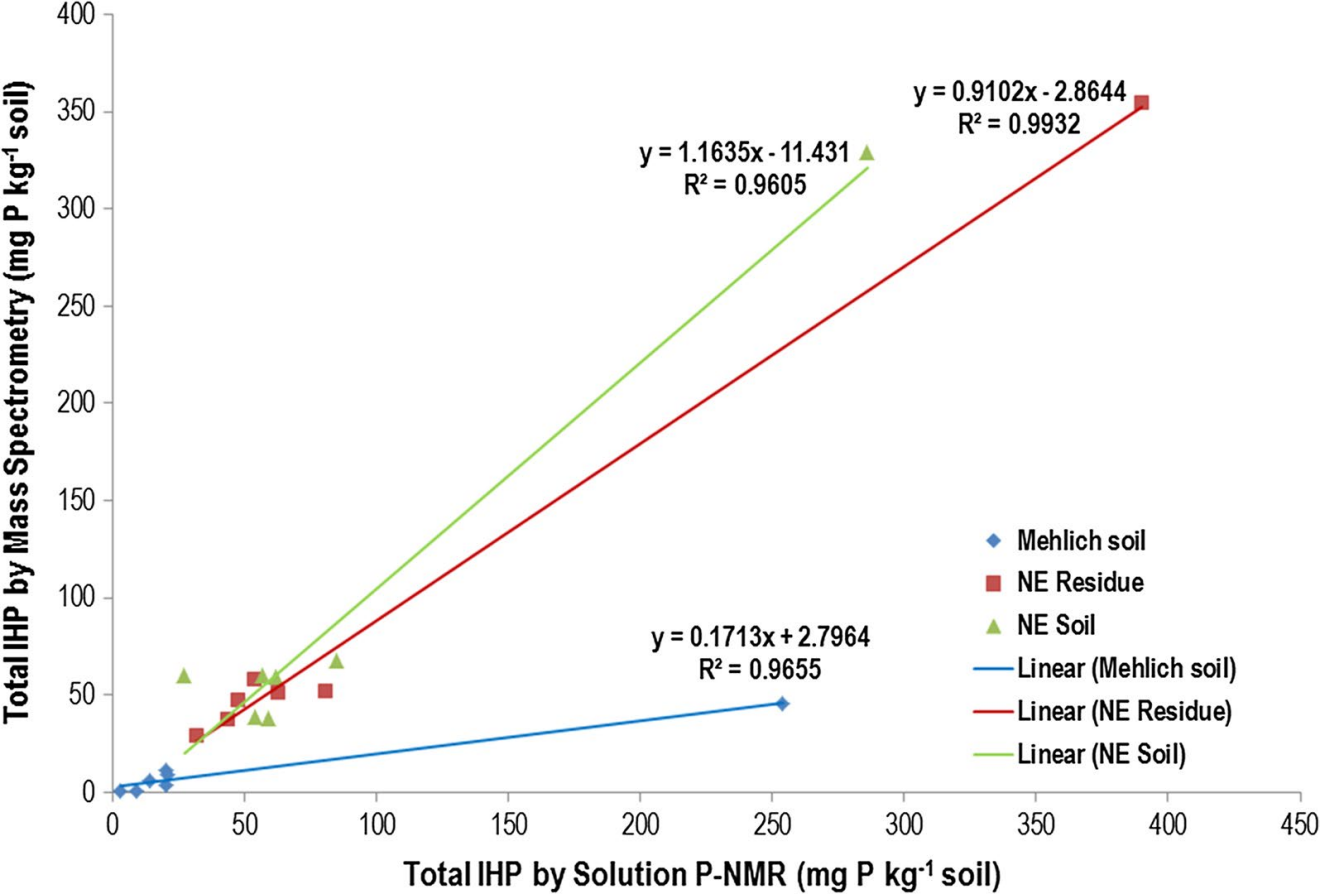
A comparison of total inositol hexaphosphate (IHP) concentrations (in mg P kg^−1^ soil) measured by solution P-NMR and mass spectrometry

Analysis of the M3 extracts by P-NMR showed that 49–87% of the extracted P was orthophosphate, with concentrations that were quite similar to those for colorimetric P (Table 5). Of the organic P compounds identified in M3 extracts by P-NMR, the majority were orthophosphate monoesters, dominated by *myo*-IHP and its stereoisomers (Tables 6, 7). Only traces of other organic P compounds were detected in the M3 extracts regardless of soil, with orthophosphate diesters comprising 6% or less of extracted P, even after correction for degradation during extraction and analysis. The presence of IHP stereoisomers was confirmed with mass spectrometry (Tables 4, 7). Although there were differences between the concentrations and percentages determined by MS and P-NMR, the results for the M3 extracts were highly related (R^2^ = 0.9655; Fig. 7). Interesting differences were also detected among the M3 extracts for the AR samples. The ratios of orthophosphate monoesters to diesters (Mono:Diester ratio, Table 6) were similar for the control, N and untreated poultry litter (PL) treatments (~ 7), but were lower than the alum-stabilized PL treatment (15.7). This reflects the much higher concentration and percentage of *myo*-IHP in the M3 extract after alum treatment, which is consistent with previous reports that alum stabilizes *myo*-IHP in poultry litter, potentially limiting its decomposition or loss in soil [15, 16, 43–45].

**Table 6:** Concentrations and percentages of organic P compounds in extracts of whole soil or residues, determined by solution P-NMR spectroscopy

	Extractant		AR-control	AR-N	AR-PL	AR-PL-alum	SK	PEI	Till Ref
Phosphonates	Mehlich III	Soil	1 (2)	2 (2)	5 (1)	5 (1)	1 (1)	2 (1)	0 (1)
	NaOH-EDTA	Residue	2 (2)	2 (1)	4 (1)	8 (1)	3 (2)	3 (1)	2 (1)
	NaOH-EDTA	Soil	2 (1)	5 (1)	5 (1)	8 (1)	5 (1)	5 (1)	3 (1)
Glucose 6-phosphate	Mehlich III	Soil	1 (1)	1 (1)	0 (0)	5 (1)	1 (1)	2 (1)	0 (1)
	NaOH-EDTA	Residue	2 (1)	2 (1)	4 (1)	8 (1)	1 (1)	3 (1)	2 (1)
	NaOH-EDTA	Soil	2 (1)	2 (1)	5 (1)	8 (1)	5 (1)	5 (1)	3 (1)
Choline phosphate	Mehlich III	Soil	1 (1)	1 (1)	0 (0)	5 (1)	1 (1)	0 (0)	0 (1)
	NaOH-EDTA	Residue	4 (1)	5 (2)	4 (1)	8 (1)	3 (1)	3 (1)	4 (1)
	NaOH-EDTA	Soil	5 (1)	4 (1)	5 (1)	8 (1)	2 (1)	5 (1)	3 (1)
Unknown 5.1 ppm	Mehlich III	Soil	1 (1)	1 (1)	3 (1)	5 (1)	2 (2)	0 (0)	0 (1)
	NaOH-EDTA	Residue	4 (1)	5 (2)	8 (1)	8 (1)	8 (4)	6 (1)	28 (9)
	NaOH-EDTA	Soil	11 (4)	9 (3)	10 (1)	25 (2)	15 (4)	11 (1)	10 (3)
Monoester 1	Mehlich III	Soil	1 (1)	1 (1)	3 (1)	5 (1)	1 (1)	2 (1)	0 (1)
	NaOH-EDTA	Residue	2 (1)	2 (1)	4 (1)	8 (1)	1 (1)	3 (1)	2 (1)
	NaOH-EDTA	Soil	2 (1)	4 (1)	5 (1)	8 (1)	5 (1)	5 (1)	3 (1)
Monoester 2	Mehlich III	Soil	2 (4)	3 (4)	8 (2)	30 (4)	6 (6)	3 (1)	2 (5)
	NaOH-EDTA	Residue	9 (4)	18 (6)	12 (2)	91 (8)	11 (5)	6 (1)	32 (10)
	NaOH-EDTA	Soil	21 (8)	18 (5)	14 (1)	41 (4)	25 (7)	16 (2)	25 (7)
Monoester 3	Mehlich III	Soil	2 (3)	2 (2)	3 (1)	5 (1)	2 (2)	2 (1)	0 (1)
	NaOH-EDTA	Residue	2 (1)	5 (1)	4 (1)	8 (1)	3 (1)	11 (3)	4 (1)
	NaOH-EDTA	Soil	5 (2)	7 (2)	10 (1)	8 (1)	5 (1)	5 (1)	5 (1)
Degradation	Mehlich III	Soil	3 (4)	3 (3)	3 (1)	15 (2)	5 (5)	3 (1)	1 (4)
	NaOH-EDTA	Residue	23 (9)	27 (9)	20 (3)	54 (5)	17 (8)	17 (4)	68 (21)
	NaOH-EDTA	Soil	25 (9)	24 (7)	26 (4)	58 (5)	45 (13)	27 (4)	27 (8)
DNA	Mehlich III	Soil	0 (0)	0 (0)	0 (0)	1 (1)	0 (0)	0 (0)	0 (0)
	NaOH-EDTA	Residue	2 (1)	2 (1)	8 (1)	1 (0)	6 (3)	4 (1)	8 (2)
	NaOH-EDTA	Soil	3 (1)	7 (2)	2 (1)	11 (1)	3 (1)	3 (0)	3 (1)
Other orthophosphate diesters	Mehlich III	Soil	1 (1)	1 (1)	2 (1)	4 (1)	1 (1)	1 (1)	1 (2)
	NaOH-EDTA	Residue	3 (1)	4 (1)	17 (3)	7 (1)	6 (3)	5 (1)	21 (6)
	NaOH-EDTA	Soil	11 (4)	10 (3)	16 (2)	21 (2)	9 (3)	8 (1)	5 (1)
Total orthophosphate monoesters	Mehlich III	Soil	27 (42)	29 (34)	37 (10)	308 (46)	21 (21)	22 (9)	6 (19)
	NaOH-EDTA	Residue	70 (27)	99 (32)	117 (20)	520 (47)	60 (30)	84 (21)	117 (36)
	NaOH-EDTA	Soil	103 (36)	129 (40)	109 (15)	385 (33)	116 (34)	133 (18)	102 (28)
	NaOH-EDTA	Difference	33 (9)	31 (7)	− 7 (− 5)	− 134 (− 15)	55 (5)	48 (− 4)	16 (− 8)
Total orthophosphate diesters	Mehlich III	Soil	4 (6)	4 (5)	5 (1)	20 (3)	6 (6)	5 (2)	2 (6)
	NaOH-EDTA	Residue	29 11)	33 (11)	45 (8)	62 (6)	29 (14)	25 (6)	96 (29)
	NaOH-EDTA	Soil	39 (14)	42 (13)	49 (7)	90 (8)	57 (17)	37 (5)	35 (10)
	NaOH-EDTA	Difference	10 (3)	9 (2)	5 (− 1)	28 (2)	28 (3)	12 (− 2)	− 61 (− 20)
Mono:Diester ratio	Mehlich III	Soil	7.2	7.1	7.0	15.7	3.3	4.4	3.0
	NaOH-EDTA	Residue	2.4	3.0	2.6	8.4	2.1	3.3	1.2
	NaOH-EDTA	Soil	2.6	3.1	2.2	4.3	2.0	3.6	2.9

Values are concentration in mg kg^−1^ (percentage of extract P). Degradation is the sum of compounds in the orthophosphate monoester region known to be products of diester degradation during analysis. Monoester 1, 2 and 3 are the sum of peak areas in these regions after subtracting the peak areas for identified P forms. Mono:Diester ratio: the ratio of orthophosphate monoesters to orthophosphate diesters. Total orthophosphate monoesters, total orthophosphate diesters, and the monoester:diester ratio were corrected for degradation. Residue is the residual soil after Mehlich III extraction. Difference is the difference in between the NaOH-EDTA extracts of whole soils and the NaOH-EDTA extracts of the residue after Mehlich III extraction

**Table 7:** Inositol hexaphosphate (IHP) stereoisomer concentrations, determined by solution P-NMR or mass spectrometry

	Extractant		AR-control	AR-N	AR-PL	AR-PL-alum	SK	PEI	Till Ref
*myo*-IHP	Mehlich III	Soil	14 (22)	12 (15)	11 (3)	219 (33)	5 (5)	7 (3)	2 (5)
NMR	NaOH-EDTA	Residue	32 (13)	40 (13)	48 (8)	336 (31)	17 (8)	32 (8)	26 (8)
	NaOH-EDTA	Soil	39 (14)	62 (19)	29 (4)	229 (19)	34 (10)	43 (6)	30 (8)
	NaOH-EDTA	Difference	7 (1)	21 (6)	− 19 (− 4)	− 107 (− 11)	18 (2)	10 (3)	3 (0)
*scyllo*-IHP	Mehlich III	Soil	4 (6)	3 (4)	3 (1)	10 (2)	1 (1)	2 (1)	1 (2)
NMR	NaOH-EDTA	Residue	4 (1)	7 (2)	4 (1)	15 (1)	7 (3)	11 (3)	9 (3)
	NaOH-EDTA	Soil	7 (3)	11 (3)	10 (3)	16 (1)	7 (2)	16 (2)	10 (3)
*neo*-IHP	Mehlich III	Soil	0 (1)	1 (1)	3 (1)	5 (1)	1 (1)	2 (1)	0 (1)
NMR	NaOH-EDTA	Residue	2 (1)	2 (1)	4 (1)	0 (0)	1 (1)	3 (1)	2 (1)
	NaOH-EDTA	Soil	2 (1)	2 (1)	5 (1)	8 (1)	2 (1)	5 (1)	3 (1)
*chiro*-IHP	Mehlich III	Soil	2 (3)	3 (4)	5 (1)	20 (3)	2 (2)	3 (1)	1 (3)
NMR	NaOH-EDTA	Residue	11 (4)	13 (4)	24 (4)	39 (4)	7 (4)	8 (2)	7 (2)
	NaOH-EDTA	Soil	9 (3)	11 (3)	20 (3)	33 (3)	15 (4)	21 (3)	13 (4)
Total IHP	Mehlich III	Soil	20 (31)	20 (24)	21 (6)	254 (38)	9 (9)	14 (6)	3 (10)
NMR	NaOH-EDTA	Residue	48 (19)	63 (21)	81 (13)	390 (35)	32 (16)	54 (14)	44 (13)
	NaOH-EDTA	Soil	57 (20)	86 (26)	62 (8)	286 (24)	59 (17)	85 (11)	54 (15)
	NaOH-EDTA	Difference	8 (1)	23 (6)	− 18 (− 5)	− 104 (− 11)	27 (2)	31 (− 3)	11 (2)
Total IHP	Mehlich III	Soil	11 (17)	4 (5)	9 (3)	46 (7)	1 (< 1)	6 (3)	1 (1)
Mass spec.	NaOH-EDTA	Residue	47 (19)	51 (15)	52 (8)	354 (32)	29 (15)	58 (15)	37 (11)
	NaOH-EDTA	Soil	60 (20)	66 (20)	59 (9)	329 (29)	38 (20)	68 (17)	39 (11)

Values are concentration in mg kg^−1^ (percentage of extract P). Residue is the residual soil after Mehlich III extraction. Total inorganic P is the sum of orthophosphate, pyrophosphate and polyphosphate. Difference is the difference in between the NaOH-EDTA extracts of whole soils and the NaOH-EDTA extracts of the residue after Mehlich III extraction

In acid soils, IHP sorption is controlled by amorphous Fe and Al oxides, while in neutral soils it will depend on calcite, clays and organic matter [46]. As such, there are likely a number of factors controlling IHP in these samples. However, there were no clear patterns of total IHP concentrations and percentages with cations in the M3 extracts. Previous reports have suggested that Fe- and Al-bound *myo*-IHP are insoluble in acid [8, 47]; however, the concentrations of total IHP were not higher in the M3 extracts of samples dominated by Ca and Mg (AR-control, AR-PL, SK) than in extracts of AR-PL-alum, although they were higher than in the Till Ref sample. These results also seem to be supported by MS analyses, where there were no differences in the profile of *myo*-IHP-cation complexes regardless of the soil cation profile. It is important to note, however, that the cations present in the M3 samples by ICP (shown in Table 3) are likely to be different from those in the samples analyzed by NMR and MS, due to the Chelex treatment and pH adjustment. It is also likely that the effects of Chelex and pH adjustment may vary for M3 extracts from different soils, or from soils with different treatments. We did not monitor the effects of these changes for the samples of this study, but recommend doing so in future research.

We were unable to detect any P forms except orthophosphate by P-NMR in initial extracts where the pH was adjusted without treatment with Chelex to remove cations, but could detect a number of P forms in all samples after Chelex treatment. This confirms the results of Jørgensen et al. [29] demonstrating that acid soil extracts could be analyzed by P-NMR after treatment to remove metals with cation exchange resins prior to pH adjustment. The differences in P forms detected with and without Chelex raise questions about previous studies that detected only orthophosphate in acid extracts of soil as part of a sequential extraction procedure for which pH was increased without using a cation exchange resin treatment [27, 28]. The results of those studies suggested that NaOH-EDTA extraction removes the majority of organic P compounds, leaving only less-soluble orthophosphate in the residues, although precipitation when preparing the sample for P-NMR was reported [22, 28]. Further investigation is warranted to confirm that no organic P forms were removed by this precipitation.

### NaOH-EDTA extraction

Extraction of whole soils with NaOH-EDTA recovered 39–97% of total soil P (Table 3), which is consistent with other studies [38]. Orthophosphate comprised 42–75% of extracted P (Table 5), which was a lower proportion of extracted P than was detected in the M3 extracts. However, the concentration of orthophosphate was higher in NaOH-EDTA extracts of whole soils than in M3 extracts for all samples. This suggests that NaOH-EDTA extracts both labile and less soluble orthophosphate, while M3 extracts only labile orthophosphate.

Higher percentages of complex inorganic P compounds (pyrophosphate and polyphosphate; Table 5) and organic P compounds were detected in the whole-soil NaOH-EDTA extracts than M3 extracts (Tables 6, 7). Concentrations of *myo*-IHP and total IHP were higher in the NaOH-EDTA extracts than in the M3 extracts for all samples. However, although *myo*-IHP and total IHP concentrations were lower in M3 than NaOH-EDTA extracts for the alum-treated PL, they constituted a higher percentage of extracted P in the M3 than in the whole soil NaOH-EDTA extract for that sample. The Mono:Diester ratio was also lower for NaOH-EDTA extracts of whole soils and residues compared to M3 extracts, for all soils (Table 6). This suggests that M3 may be preferentially extracting orthophosphate monoesters from samples compared with NaOH-EDTA, which extracts a wider range of P forms. The concentrations of total organic P determined by NMR were lower than those determined by the ignition method in whole soils (Table 1). However, the percentage of organic P determined by the two methods was similar, which also indicates that NaOH-EDTA extracts a representative range of soil organic P forms.

In the AR samples, the initial soils were the same, but 20 years of treatment altered P cycling in the soils. The three treatments without alum (AR-control, AR-N and AR-PL) were generally similar to one another, but different from the alum-stabilized poultry litter treatment. One notable difference was in *myo*-IHP for the NaOH-EDTA whole-soil extracts, where concentration and proportion of *myo*-IHP were much lower in the AR-PL sample than for the AR-control and AR-N soils. This indicates that the *myo*-IHP added in untreated poultry litter for 20 years may be mineralized to other P forms, or lost from the soil in runoff or leaching [21]. This does not appear to be happening for the poultry litter stabilized by alum. Other research [48] provided strong evidence that IHP reacted with amorphous Al-hydroxide to form an Al-IHP precipitant, reducing the solubility, lability, and availability of IHP to further transformations or mineralization.

Extracting the M3 residues with NaOH-EDTA recovered P concentrations and proportions that were similar to the whole soil NaOH-EDTA extracts for the AR-control, AR-N, and Till Ref samples, but lower than the whole soil NaOH-EDTA extracts for the AR-PL, AR-PL-alum, SK and PEI soils (Table 3). The NaOH-EDTA extracts of residues contained a range of P compounds that were comparable to the whole-soil NaOH-EDTA extracts. However, the differences between the whole soil and residues varied among samples with respect to changes in concentrations. Orthophosphate and total inorganic P concentrations were higher in residues for the AR-control and AR-N soils, but were lower for the other soils (Table 5). Recoveries of total P in the whole soil NaOH-EDTA extracts were 59 and 62%, respectively for these samples. The NaOH-EDTA extraction targeted organic P over inorganic P compounds, and the unextracted P after NaOH-EDTA is thought to be poorly soluble orthophosphate [22], although as noted this warrants further investigation. Pre-extraction with an acid-EDTA solution (M3), which complexes cations, may alter the solubility of inorganic P, which is then extracted by NaOH-EDTA.

For most of these samples, the calculated differences in concentrations of organic P compounds (total orthophosphate monoesters and diesters; Table 6) between extracts of whole soils and extracts of residues were positive (> 0). This suggests that comparing NaOH-EDTA extracts before and after other extractants can give information about changes in P compounds. Additionally, it can yield information about how those P compounds are stabilized in soils (e.g. before and after oxalate extraction; [29]) or the potential reactivity of those P compounds in the environment, depending on the pre-extractant. This can also be used to fully understand or refine more commonly used P extractants, as was done for the M3 method here.

In addition to the M3 results, the concentrations of total IHP determined by MS aligned well with those determined by P-NMR in the NaOH-EDTA extracts (Table 7, Fig. 7). The MS concentrations were typically in agreement (R^2^ = 0.9605 for whole soil; R^2^ = 0.9932 for residues). For most of these samples, the peaks for IHP stereoisomers were well-resolved in P-NMR spectra (Fig. 5), giving us confidence in the concentrations determined by P-NMR. This suggests that while the MS and NMR results are similar, MS results must include IHP adducts that are prevalent in NaOH-EDTA extracts. Several studies have shown that the complex formation constants of both *myo*-IHP and EDTA are quite high, and in some cases both can compete for cations [49–51] in the matrix, altering the mass of the *myo*-IHP to something that was not detected. There are several peaks evident in Fig. 6 that show *myo*-IHP complexed with different cations even after Chelex treatment and the addition of 0.5 mol L^−1^ EDTA. It is evident that there is a clear equilibrium between IHP and EDTA, even when there is excess EDTA [52, 53]. The most abundant peak is at 339.91 (Na complex), followed by 600.87, 680.84 (Mg complexes), and 279.93 (NaK complex), which are shown in Table 4. Further investigation is needed to determine if the different IHP stereoisomers form complexes with varying stability constants. While the direct injection method removes the necessity for chromatography, there is no way to separate the stereoisomers. Organic phosphates are ideal candidates for ion chromatography due to the easily ionized phosphate moiety. Techniques for MS have been developed to look at the different inositol phosphates in solution; however, these methods must be adapted to accommodate complex soil extraction matrices [40]. These results nevertheless support the NMR results showing a broad range of P compounds in various soils and help to explain the discrepancy between colorimetry and ICP results.

### Implications with respect to phosphorus management

The results of this study clearly show that very different P concentrations can be measured in M3 extracts if analyzed by colorimetry or ICP, depending on the sample. Similar P concentrations were determined by ICP (TP, Table 3) and colorimetrically (MRP, Table 5) for the Till Ref sample (30 and 29 mg kg^−1^), PEI (244 and 211 mg kg^−1^) and AR-PL (382 and 378 mg kg^−1^) samples, while higher concentrations were determined by ICP for the AR-control (64 and 31 mg kg^−1^), AR-N (84 and 58 mg kg^−1^), AR-PL-alum (675 and 369 mg kg^−1^) and SK (99 and 60 mg kg^−1^). For the samples with higher ICP P concentrations, those concentrations were 48–69% higher than P measured colorimetrically. This supports the view that M3 P measured colorimetrically and M3 P measured by ICP should be considered to be different soil tests, using different guidelines for fertilizer recommendations, including field validation for each method [10, 13]. However, it should also be noted that some of these differences fall within the range of error expected for M3 analysis, for which small changes in protocol can alter results [42]. As such, small differences between ICP and colorimetric measurements will have little effect on fertilizer recommendations for many soils.

These results show that ICP analysis of M3 extracts includes both inorganic and organic P compounds, with *myo*-IHP as the predominant organic P form extracted from all tested soils. Although labile organic compounds are likely to contribute to plant-available P during the growing season, and are not adequately addressed by current soil tests [5], further research is needed to determine how much of the organic P in these extracts contributes to plant-available P in the growing season. Some studies have reported the mineralization of *myo*-IHP [45, 47]; however, this will vary among soils. In soils with neutral pH, *myo*-IHP is associated with calcite, organic matter and metal oxides. However, as soil pH decreases, *myo*-IHP sorption will increase as the sorption capacity of metal oxides increases [29, 46]. Agricultural practices such as liming or fertilization can alter soil pH, in turn affecting sorption capacity. A recent review of P-NMR studies in cropland soils indicated that orthophosphate, but not *myo*-IHP, varied with P fertilization and was lowest in soils receiving the least fertilizer for several studies [54]. This suggests that colorimetric analysis, measuring orthophosphate concentrations in M3 extracts, may be more reliable with respect to predicting crop fertilizer response, particularly across a wider range of soil types and management practices. It may be possible to determine an indicator that could be measured during ICP analysis of M3 extracts to flag samples that might be high in MUP, such as the concentration of a particular cation or the ratio of two or more cations. However, this will require further testing with a greater range of samples than was used for the current study.

In many regions, the M3 test has been used as an environmental P test to assess P loss potential, based on good correspondence between soil P sorption saturation estimated by M3 to other methods [18, 19], and P saturation equations have been developed from M3 extracts to set P application cutoffs for use in P index development across regional boundaries [e.g. 55]. Phosphorus sorption capacity is usually determined using inorganic P (KH_2_PO_4_), and is rarely tested for organic P compounds. However, a test comparing the sorption and desorption of *myo*-IHP and orthophosphate on reference minerals such as goethite clearly demonstrated different sorption capacities for these P compounds, which varied depending on the mineral [21]. This suggests that P sorption will be even more complex in soils containing a number of different P compounds and a range of minerals. Furthermore, it is supported by the variability in *myo*-IHP concentrations determined in the current study in M3 extracts from soils with different management practices on the same soils. The complex interaction between P, soil cations, and pH should be more closely considered in terms of management, especially in amended soils. Agricultural lands receiving manures as well as pH amendments have large pools of organic P that can be seen in the M3 soil test. However, many of the long-term agronomic effects of organic P accumulation in amended soils are not well studied and may become important in the future. Different criteria may need to be developed for different soils, depending on soil properties and management practices [55]. It is also important to remember that the assessment of P loss potential should not rely on soil P testing alone, and must consider a number of factors including transport processes, management practices, and potentially multiple soil tests [2, 6].

The clear differences in *myo*-IHP concentrations in M3 extracts of the alum-treated and untreated AR soils fertilized with poultry litter suggests that more research is needed to determine the long-term implications of the widely-used practice of alum treatment. Although alum treatment has been shown to reduce water-extractable P compared to untreated litter [15, 16], the build-up of high concentrations of *myo*-IHP in soils may affect soil fertility over time. Research is also needed to determine any possible adverse effects from the transport of alum-stabilized *myo*-IHP to water bodies, for example by erosion.

## Conclusions

This study used P-NMR and mass spectrometry to characterize the P forms removed from soils by the Mehlich III (M3) soil extractant. The results of this study demonstrate that M3 extracts organic P compounds from soils in addition to orthophosphate, with *myo*-IHP (phytate) the predominant organic P form in M3 extracts for all soils used in this study. However, the concentrations of organic P varied among soil samples, including long-term fertilizer treatments on the same soils. For most soils, analysis of M3 extracts by ICP produced higher P concentrations than colorimetric analysis, reflecting organic P in the M3 extracts, but this was not consistent for all samples. The M3 soil test is widely used in North America to develop fertilizer recommendations and to develop P indices to control P loss in runoff. Due to the variability of organic P in M3 extracts, basing soil P recommendations or indices on M3 extracts analyzed by ICP alone may produce erroneous P values. Further testing is required to develop an indicator to screen ICP-analyzed M3 extracts for high MUP, such as cation concentrations or ratios of cation. These results have important implications for the field of soil P management, given with widespread use of this extractant.

The results of this study also showed that treating acid extracts with a cation exchange resin prior to adjusting the pH to the range required for optimal P-NMR analysis preserved organic P compounds that might otherwise be removed from solution by precipitation. This technique could be used to expand the extraction procedures for soil P-NMR. Comparing NaOH-EDTA extracts of whole soils with extracted residues after various pre-treatments could also provide information that will be useful for understanding P cycling in soils, or to refine widely used techniques (such as other soil test P extractions. This study also demonstrated that techniques such as P-NMR and MS should be seen as complimentary, each yielding additional information that analysis by a single technique may not provide. Analysis with a series of techniques and spectroscopic methods may be the most appropriate way to increase the usefulness of information that is gained from simple soil tests.

## Abbreviations

D_2_O: deuterium oxide
DNA: deoxyribonucleic acid
EDTA: ethylenediaminetetraacetic acid
ICP: inductively coupled plasma-based spectrometry
IHP: inositol hexaphosphate
M3: Mehlich III soil test
MRP: molybdate-reactive P
MS: mass spectrometry
MUP: molybdate unreactive P
P-NMR: ^31^P nuclear magnetic resonance spectroscopy
